# Integrated Multi‐Omics Analyses Reveal Causal DNA Methylation‐Breast Cancer Associations Mediated by Plasma Proteins

**DOI:** 10.1111/jcmm.71424

**Published:** 2026-11-08

**Authors:** Wei Wang, Ziye Cheng, Ruidong Liu, Xufeng Cheng, Hanghang Chen

**Affiliations:** ^1^ Breast Surgery The First Affiliated Hospital of Henan University of Traditional Chinese Medicine Zhengzhou China; ^2^ School of Traditional Chinese Medicine Beijing University of Chinese Medicine Beijing China

**Keywords:** breast cancer, DNA methylation, Mendelian randomization, plasma proteins

## Abstract

Although aberrant DNA methylation is implicated in breast cancer (BC) pathogenesis, establishing causality is difficult and the downstream mediators are unclear. We investigated the causal role of blood DNA methylation and its proteomic mediators. Differentially methylated positions (DMPs) were identified from a genome‐wide blood methylation dataset, and two‐sample Mendelian randomization (MR) was performed using methylation quantitative trait loci for the significant DMPs together with BC genetic data from two independent consortia (FinnGen and the Breast Cancer Association Consortium), applying inverse‐variance weighted and generalized summary‐data‐based MR with validation by Bayesian colocalization and PheWAS. Proteome‐wide mediation and single‐cell RNA sequencing (scRNA‐seq) analyses explored downstream pathways. We identified 19,169 DMPs, and MR revealed 95 CpG sites with consistent, MR‐supported associations with BC. Meta‐analysis confirmed three high‐confidence loci (cg15146122, OR = 0.91; cg01529201, OR = 1.14; cg24704912, OR = 1.11), with colocalization support for two of them. Mediation analysis identified 203 bc‐associated proteins, the three CpG sites influencing 41‐314 proteins involved in immune regulation, apoptosis and lysosomal pathways and scRNA‐seq localized the mediator proteins to specific cell populations of the tumour microenvironment. Blood DNA methylation is therefore associated with BC risk in an MR‐supported manner through specific plasma proteins, highlighting candidate epigenetic biomarkers.

**Abbreviations**
BC: breast cancer
BCAC: Breast Cancer Association Consortium
CI: confidence interval
CpG: cytosine‐phosphate‐guanine
DMPs: differentially methylated positions
DNAm: DNA methylation
eQTL: expression quantitative trait locus
FDR: false discovery rate
GoDMC: Genetics of DNA Methylation Consortium
GSMR: Generalized Summary‐data‐based Mendelian Randomization
GWAS: Genome wide association study
IV: instrumental variable
IVW: inverse variance weighted
LD: linkage disequilibrium
MAF: minor allele frequency
meQTL: methylation quantitative trait locus
MR: Mendelian randomization
MR‐PRESSO: Mendelian Randomization Pleiotropy RESidual Sum and Outlier
OR: odds ratio
PheWAS‐MR: Phenome‐wide Mendelian randomization
PP.H4: posterior probability for hypothesis 4 (colocalization analysis)
pQTL: protein quantitative trait locus
scRNA‐seq: single‐cell RNA sequencing
SNP: single nucleotide polymorphism
TSMR: two‐sample Mendelian randomization
UKB‐PPP: UK Biobank Pharma Proteomics Project

## 1 Introduction

Breast cancer (BC) persists as a major global health challenge, representing the most frequently diagnosed malignancy and a leading cause of cancer‐related mortality among women worldwide [1]. The rising incidence, especially in developing nations, coupled with the limitations of current screening methods—such as suboptimal sensitivity and high false‐positive rates [2]—highlights the urgent need for better risk stratification and early detection strategies [3]. DNA methylation has emerged as a pivotal epigenetic mechanism at the interface of genetic predisposition and environmental exposures, influencing cancer risk. Aberrant DNA methylation patterns are recognized as potential biomarkers for cancer diagnosis and prognosis.

DNA methylation, an epigenetic modification involving the addition of a methyl group to cytosine bases in CpG dinucleotides, regulates gene expression without altering the DNA sequence itself. In BC specifically, such aberrant methylation—particularly promoter hypermethylation of tumour‐suppressor genes—can silence critical regulators of cell cycle control, DNA repair and immune surveillance, thereby facilitating tumour initiation and progression [4, 5, 6, 7, 8, 9]. While numerous studies have associated specific methylation changes with BC development and drug resistance, the majority of evidence remains correlative, derived from observational studies susceptible to confounding and reverse causation. A comprehensive, causally oriented investigation into the genome‐wide landscape of DNA methylation in BC susceptibility is therefore necessary to distinguish driving epigenetic events from mere consequences of the disease.

Mendelian randomization (MR) utilizes genetic variants, such as meQTLs, as instrumental variables to infer causal relationships between exposures and disease outcomes. This method minimizes confounding and reverse causation, as genetic alleles are randomly assigned at conception. In the context of BC, MR has begun to validate the causal roles of specific epigenetic traits—including smoking‐ and alcohol‐related DNA methylation marks—in disease aetiology [10, 11, 12]. Recent applications of MR have begun to validate the causal roles of various molecular traits in disease aetiology [13, 14, 15]. Nevertheless, while some studies have linked genetic variants to both methylation and disease risk [10, 11], a systematic, large‐scale MR analysis specifically focused on identifying causal CpG sites and delineating their effects through mediating pathways like the plasma proteome is still lacking. Furthermore, the integration of single‐cell transcriptomics to pinpoint the cellular context of these mediating proteins within the tumour microenvironment represents a significant advance towards understanding the mechanistic pathway from epigenetic variation to disease phenotype.

Here, we present a multi‐omics study investigating the potential causal relationship between blood DNA methylation and BC risk. We identified differentially methylated positions (DMPs) from peripheral blood methylation profiles and applied a two‐sample MR framework, integrating methylation quantitative trait loci (meQTLs) from GoDMC with BC genome‐wide association study (GWAS) data from BCAC and FinnGen, to infer genetically proxied associations. Significant findings were validated via sensitivity analyses, Bayesian colocalization and proteome‐wide MR to identify plasma protein mediators. Single‐cell RNA sequencing characterized mediator expression within the breast tumour microenvironment. Our study identifies robust, MR‐supported links between DNA methylation and BC, providing novel insights into the disease's epigenetic architecture and highlighting candidate biomarkers for risk assessment and early detection.

## 2 Materials and Methods

### 2.1 Ethics

This study used only publicly available, de‐identified data, comprising summary‐level GWAS and QTL statistics and open‐access Gene Expression Omnibus datasets. Ethical approval and written informed consent for each original cohort were obtained by the investigators of the corresponding studies, and all analyses were conducted in accordance with the Declaration of Helsinki. No additional ethical approval or informed consent was required for this secondary analysis.

### 2.2 Data Sources

To investigate differential blood DNA methylation patterns in BC, we utilized high‐quality data from the Gene Expression Omnibus (GEO) accession GSE104942 [5], which comprises peripheral blood DNA methylation profiles. This dataset employs the Illumina Infinium HumanMethylation450K BeadChip—the most widely adopted microarray platform in population‐scale epigenome‐wide association studies—providing robust, genome‐wide quantification of 485,577 CpG sites with established reproducibility. DNA methylation was measured from peripheral blood samples of 87 histologically confirmed BC cases and 123 rigorously screened healthy controls, all processed uniformly to minimize batch effects. DNAm levels are reported as standardized beta‐values (*β* = M/(M + U + 100), where M and U represent methylated and unmethylated probe intensities), ensuring consistent biological interpretation across loci.

We sourced methylation quantitative trait loci (meQTLs) for exposures from the definitive Genetics of DNA Methylation Consortium (GoDMC) database [16]. GoDMC represents a major international consortium effort, providing the largest harmonized summary statistics for DNA methylation quantitative trait loci. Its rigorous meta‐analysis integrates data from 420,509 CpG sites across 27,750 individuals of European ancestry derived from 36 independent cohorts. This exceptional scale and diversity ensure high statistical power and minimize cohort‐specific biases. The consortium employs stringent quality control and standardized analytical pipelines across contributing studies, guaranteeing data integrity. Leveraging this authoritative resource provides robust instrumental variables, significantly enhancing the reliability and generalizability of our causal inference for DNA methylation effects.

To investigate the mediating role of plasma proteins, we utilized protein quantitative trait loci (pQTLs) from the UK Biobank Pharma Proteomics Project (UKB‐PPP) study, a landmark analysis embedded within the UK Biobank. This resource provides comprehensive data on 2940 plasma proteins measured using a high‐throughput Olink proteomics assay in 33,628 UK Biobank participants of European ancestry. The unprecedented scale of this dataset, coupled with its integration with rich genetic and health record data from the UK Biobank, establishes it as a premier resource for proteomic research. The rigorous standardization and quality control procedures employed ensure high data fidelity.

Summary‐level data for BC risk were obtained from two of the most authoritative genome‐wide association study (GWAS) consortia: the Breast Cancer Association Consortium (BCAC) [17] and the FinnGen study (Release 11) [18]. The BCAC is the largest and most comprehensive genetic association resource for BC, comprising over 247,173 individuals of European ancestry (133,384 cases and 113,789 controls) from 100 studies, providing exceptional power to detect genetic associations, including for specific tumour subtypes. The FinnGen study integrates genomic data with national health registries from over 222,080 Finnish participants, offering a unique population‐based design that minimizes confounding and enhances phenotyping accuracy. The exclusive use of European‐ancestry individuals across exposures and outcomes minimizes bias from population stratification. This dual‐source approach for the outcome significantly bolsters the robustness, generalizability, and validity of our causal inference estimates.

To precisely localize the expression of mediator proteins within the breast tissue microenvironment, we analysed single‐cell RNA sequencing (scRNA‐seq) data from Gene Expression Omnibus accession GSE161529 [19]. This dataset profiles 421,761 cells from 52 human samples, including both tumour and normal adjacent tissue from BC patients. Generated using 10× Genomics technology, it provides high‐resolution expression profiles across diverse cell types. A major strength of this resource is the direct comparison of malignant and non‐malignant ecosystems from the same patients, effectively controlling for inter‐individual variation. This enables the unambiguous identification of cell type‐specific gene expression for our candidate mediator proteins within the tumour microenvironment, a critical advantage over bulk tissue analyses. The public availability of this data facilitates robust validation of our findings. All data sources, original publications and information used in this study are detailed in Table S1.

### 2.3 DMPs Analysis

We conducted normalization and differential methylation analysis using established R packages. Missing beta values were imputed via k‐nearest neighbour (KNN) imputation [20] using the impute R package. Probes with mean beta values below 0.005 across all samples were removed to exclude poorly performing loci. Beta‐value quantile normalization was then applied using the betaqn function from the wateRmelon R package [21], which performs stratified quantile normalization across probe types, thereby correcting the distinct signal distributions of Type I and Type II Infinium probes and mitigating technical variation. The normalized beta matrix was converted to a GenomicRatioSet using the minfi package [22]. Differential methylation analysis was conducted using the dmpFinder function, which fits a linear model on *M*‐values (logit‐transformed beta values)—a transformation that further stabilizes variance across probe types—comparing healthy controls and BC patients with an *F*‐test. *p*‐values were adjusted for multiple testing using the false discovery rate (FDR) correction, with differentially methylated positions (DMPs) defined at *q* < 0.05.

### 2.4 Two‐Sample MR Analysis

Instrumental variable (IV) selection was performed with stringent criteria to ensure robustness. Single‐nucleotide polymorphisms (SNPs) significantly associated with each DMP at a genome‐wide significance threshold (*p* < 5 × 10^−8^) were selected as potential IVs. To ensure independence, we performed linkage disequilibrium (LD)‐based clumping (*r* ^2^ < 0.001 within a 10,000 kb window) using the 1000 Genomes Project Phase 3 European reference panel to account for population‐specific LD structure. The strength of each IV was assessed by calculating the *F*‐statistic using the formula *F* = (beta/se)^2^. Conventionally, IVs with an *F*‐statistic greater than 10 are considered strong, mitigating bias from weak instruments [23]; all IVs with an *F*‐statistic below this threshold were excluded. Furthermore, to prevent horizontal pleiotropy via the outcome, we rigorously excluded any IVs that were directly associated with BC at a suggestive significance level (*p* < 1 × 10^−5^).

We conducted the two‐sample MR analysis using the ‘TwoSampleMR’ package [24] in R. The inverse variance weighted (IVW) method, which provides the highest statistical power under the assumption that all genetic variants are valid instruments, served as our primary analytical approach [25]. In instances where only a single SNP was available for a CpG site, the Wald ratio method was applied [26]. Prior to analysis, all SNPs were harmonized to ensure the same effect allele was referenced for both the exposure and outcome datasets. MR estimates were then computed using the ‘mr’ function.

We employed Cochran's *Q* statistics to assess heterogeneity [27] and the MR‐Egger method, including its intercept term, to examine directional pleiotropy [28] in the harmonized data. *p* > 0.05 was considered of no heterogeneity or pleiotropy. Horizontal pleiotropy was further interrogated with MR‐PRESSO [29], which combines a global test for overall pleiotropy, an outlier test that detects and removes pleiotropic variants, and a distortion test that compares the causal estimate before and after outlier removal, thereby yielding an outlier‐corrected estimate; the default settings of the MRPRESSO R package were used. Additionally, we utilized the MR Steiger directionality test to exclude potential reverse causalities [30].

### 2.5 GSMR Analysis

To enhance the robustness of our causal inference and directly address the critical issue of pleiotropy, we further applied the Generalized Summary‐data‐based Mendelian Randomization (GSMR) method. GSMR (gsmr2, version 1.1.1) extends the standard IVW framework with a heterogeneity‐based model selection procedure, the HEIDI (Heterogeneity in Dependent Instruments) test, which evaluates whether the genetic instruments are consistent with a single causal variant. GSMR was applied with the instrument selection described above (*p* < 5 × 10^−8^ for SNP significance and LD *r* ^2^ < 0.05 for pruning within 10,000 kb using 1000 Genomes European reference data). Two aspects of the configuration used here should be stated explicitly. First, the HEIDI‐outlier filtering was run with a permissive multi‐SNP HEIDI threshold (5) and therefore neither flagged nor removed any instrument. Second, GSMR was used as an independent replication of the TSMR estimates rather than as a pleiotropy‐robustness filter, and pleiotropy was assessed separately with the MR‐Egger intercept and Cochran's *Q* statistics; concordant effect directions between GSMR and TSMR are therefore reported as replication and not as evidence against horizontal pleiotropy.

### 2.6 Meta‐Analysis

To enhance the reproducibility and robustness of our findings, we first identified CpG sites exhibiting consistent causal effects in both the TSMR and GSMR analyses; GSMR was treated as an independent replication of TSMR rather than being pooled together with it, to avoid combining non‐independent estimates derived from the same underlying GWAS summary statistics. For the CpG sites surviving both methods, the TSMR causal estimates obtained from the two genetically independent, non‐overlapping GWAS consortia—FinnGen R11 and the Breast Cancer Association Consortium (BCAC)—were subsequently meta‐analysed to combine statistically independent evidence. The meta‐analysis was implemented using the ‘meta’ R package (version 6.2‐1) [31]. Heterogeneity across the two cohort‐specific estimates was quantitatively assessed using Cochran's *Q* test and Higgins's *I* ^2^ statistic. Consistent with established practices, significant heterogeneity was defined a priori by a *Q*‐test *p*‐value of less than 0.05 or an *I* ^2^ value exceeding 50% [32]. A random‐effects model was applied when substantial heterogeneity was detected; otherwise, a fixed‐effects model was used as the primary method to ensure robust pooled estimates.

### 2.7 Colocalization Analysis

To further validate the causal associations identified through MR and meta‐analysis, we employed a Bayesian colocalization analysis using the ‘coloc’ R package [33]. This method examines whether DNA methylation at significant CpG sites and BC risk share a common causal genetic variant within a defined genomic region, thereby reducing confounding by linkage disequilibrium. We analysed *a* ± 1 Mb window centred on each CpG, using default prior probabilities (p1 = 1e‐4, p2 = 1e‐4, p12 = 1e‐5) as previously established [34]. Colocalization evidence was evaluated based on the posterior probability for hypothesis 4 (PP.H4), which indicates that both traits are driven by a single shared causal variant. A PP.H4 > 0.8 was considered strong evidence for colocalization, strengthening confidence that the observed MR association is not due to independent genetic signals in close proximity.

### 2.8 PheWAS Analysis

To identify potential pleiotropic effects of the SNPs serving as instrumental variables (IVs) for the CpG sites, we conducted a comprehensive Phenome‐Wide Association Study (PheWAS) analysis using the PheWeb platform (https://pheweb.org/). This analysis systematically interrogated the association of each IV with a broad spectrum of 1419 complex traits and diseases. We applied a stringent significance threshold (*p* < 1 × 10^−5^) to flag SNPs demonstrating associations with phenotypes other than BC. This rigorous, large‐scale screening is a major strength of our approach, as it effectively identifies horizontal pleiotropy—where a genetic variant influences the outcome via pathways independent of the exposure. By excluding pleiotropic IVs, we significantly enhance the specificity of our Mendelian randomization estimates and strengthen the causal inference for the relationship between DNA methylation and BC risk.

### 2.9 Proteome‐Wide Mediation Analysis

To investigate the potential mechanism by which DNA methylation influences BC risk through protein expression, we performed a TSMR analysis examining the causal relationships between significant CpG sites and 2940 plasma proteins from the UKB‐PPP database. Results were visualized using Manhattan plots, which highlighted proteins with significant causal associations after multiple testing correction.

Proteins demonstrating significant causal associations with BC risk were subsequently subjected to functional enrichment analysis. Gene Ontology (GO) terms and Kyoto Encyclopedia of Genes and Genomes (KEGG) pathways were analysed using the ‘clusterProfiler’ R package [35]. This step aimed to interpret the biological functions (Biological Process, Cellular Component, Molecular Function) and signalling pathways (e.g., cytokine signalling, immune regulation) potentially involved in BC pathogenesis, providing mechanistic insights into the methylome‐proteome‐disease axis.

Finally, a two‐step MR mediation analysis was conducted to quantify the potential mediating effects of significant proteins in the pathway from CpG methylation to BC risk. In the first step, we assessed the causal effect of the CpG on the protein; in the second step, we evaluated the causal effect of the same protein on BC. The mediation proportion was calculated as (*β*_CpG→Protein_ × *β*_Protein→BC_)/*β*_CpG→BC_. This approach allowed us to disentangle the direct effect of methylation from the indirect effect mediated through specific plasma proteins, strengthening the inference of a plausible biological pathway.

### 2.10 Chromosomal Visualization of Causal Loci

We generated a chromosomal map to illustrate the genomic positions of causal CpG sites and their associated mediator proteins using PhenoGram plot, a specialized tool for visualizing phenotype‐genome associations. The GRCh37/hg19 genome assembly was used as the reference. Causal CpGs and protein‐coding genes were plotted along the chromosomes, with distinct colours and shapes assigned to differentiate between methylation sites and protein loci. This visualization provides an intuitive overview of the genomic distribution, helping to identify potential co‐localization or clustering of causal elements in specific chromosomal regions, thereby supporting the biological plausibility of the identified associations.

### 2.11 Validation of Causal CpG Methylation Levels

To experimentally validate the functional relevance of the identified causal CpGs, we quantified their methylation levels in a DNA methylation dataset comprising both healthy controls and BC patients. Methylation beta‐values (ranging from 0 to 1) for the specific CpG sites were extracted from the dataset. The differences in methylation levels between the healthy control group and the BC group were assessed using the Wilcoxon rank‐sum test. The results were visualized using boxplots, which clearly displayed the median, quartiles and potential outliers of the methylation distribution for each group, providing an intuitive graphical representation of the hypomethylation or hypermethylation status of the causal CpGs in BC pathogenesis. This analysis provided direct empirical evidence supporting the regulatory role of these epigenetic marks.

### 2.12 Single‐Cell Expression Analysis of Mediator Proteins

To elucidate the cell type‐specific expression patterns of the candidate mediator proteins within the breast tumour microenvironment, we performed a detailed analysis of single‐cell RNA sequencing data using the standard Seurat workflow. Quality control was applied in two stages. In a first, per‐sample step, genes detected in fewer than three cells were discarded (min.cells = 3) and cells with fewer than 300 detected genes were discarded (min.features = 300). After the samples were merged, cells with fewer than 300 detected genes (nFeature_RNA < 300) or with more than 20% of counts mapping to mitochondrial genes (percent.mt < 20, computed with PercentageFeatureSet(pattern = ‘^MT‐’) on the GRCh38 reference) were removed. These thresholds retained 271,532 cells and 28,273 genes across the 47 samples that passed quality control (normal, 13 samples; ER+, 19; HER2+, 6; PR+, 1; TNBC, 8); the median number of detected genes per cell was 1130 (range 301–10,660) and the median mitochondrial count fraction was 5.46% (range 0%–20%). Counts were normalized with LogNormalize (scale factor 10,000) and 2000 highly variable genes were selected using the variance‐stabilizing transformation. All single‐cell analyses were run in R 4.2.3 with Seurat 4.4.0 and harmony 1.2.1; random seeds were fixed and are documented in the accompanying analysis code. Critical steps included the removal of potential doublets—artificial droplets containing two cells that can generate misleading hybrid expression profiles—using the DoubletFinder package. This process involved parameter optimization (e.g., selecting the optimal pK value) and homotypic doublet proportion estimation to accurately identify and exclude these technical artefacts, thereby ensuring the reliability of subsequent cell clustering and analysis. Following doublet removal, the data were scaled, 50 principal components were computed, and batch effects were corrected with Harmony (theta = 2, maximum 10 iterations); the first 10 principal components were used for graph‐based clustering with the Louvain algorithm at resolution 1, and the results were visualized in two dimensions using UMAP (n.neighbors = 30, min.dist = 0.3). Cell types were meticulously annotated based on the expression of well‐established canonical marker genes validated in our previous study [36]. Finally, to precisely compare the expression of the target protein‐coding genes across different cell states, we generated a dot plot, which simultaneously visualizes the percentage of cells expressing each gene and the average expression level within each annotated cell cluster for both healthy control and BC groups.

## 3 Results

### 3.1 Genome‐Wide Differential Methylation Analysis in BC

We identified 19,169 DMPs between BC patients and healthy controls (*q*‐value < 0.05) using the minfi package. Raw methylation data underwent rigorous preprocessing: background correction with noob normalization, probe‐type bias adjustment and removal of cross‐reactive probes. Differential analysis was performed via the dmpFinder function, which employs a linear model (empirical Bayes moderated *t*‐test) to compare beta‐values across groups. Among these DMPs, 14,118 exhibited hypermethylation and 5051 showed hypomethylation in BC patients. Full results are catalogued in Table S2.

### 3.2 Identification of Cross‐Validated Causal CpG Sites for BC via TSMR

Utilizing the 19,169 differentially methylated CpGs, we performed a TSMR analysis to infer causal relationships between blood‐based DNA methylation and BC risk. Genetic instruments for each CpG were selected as meQTLs from the GoDMC database (*p* < 5 × 10^−8^, LD clumping *r* ^2^ < 0.001, *F*‐statistic > 10). We integrated these instruments with GWAS summary statistics from two independent cohorts: FinnGen (R11) and the BCAC. The analysis identified 705 CpGs significantly associated with BC risk in FinnGen (IVW FDR < 0.05) and 872 in BCAC, visualized using Manhattan plots (Figure 1A,B) that highlight chromosomal distributions of causal signals. Intersection of these sets yielded 95 high‐confidence CpGs consistently causal across both cohorts, suggesting robust biological relevance. Sensitivity analyses, including MR‐Egger regression and Cochran's *Q* test, confirmed minimal pleiotropy or heterogeneity. Full TSMR results, including beta coefficients, standard errors and *p*‐values, are catalogued in Table S3.

**FIGURE 1:**
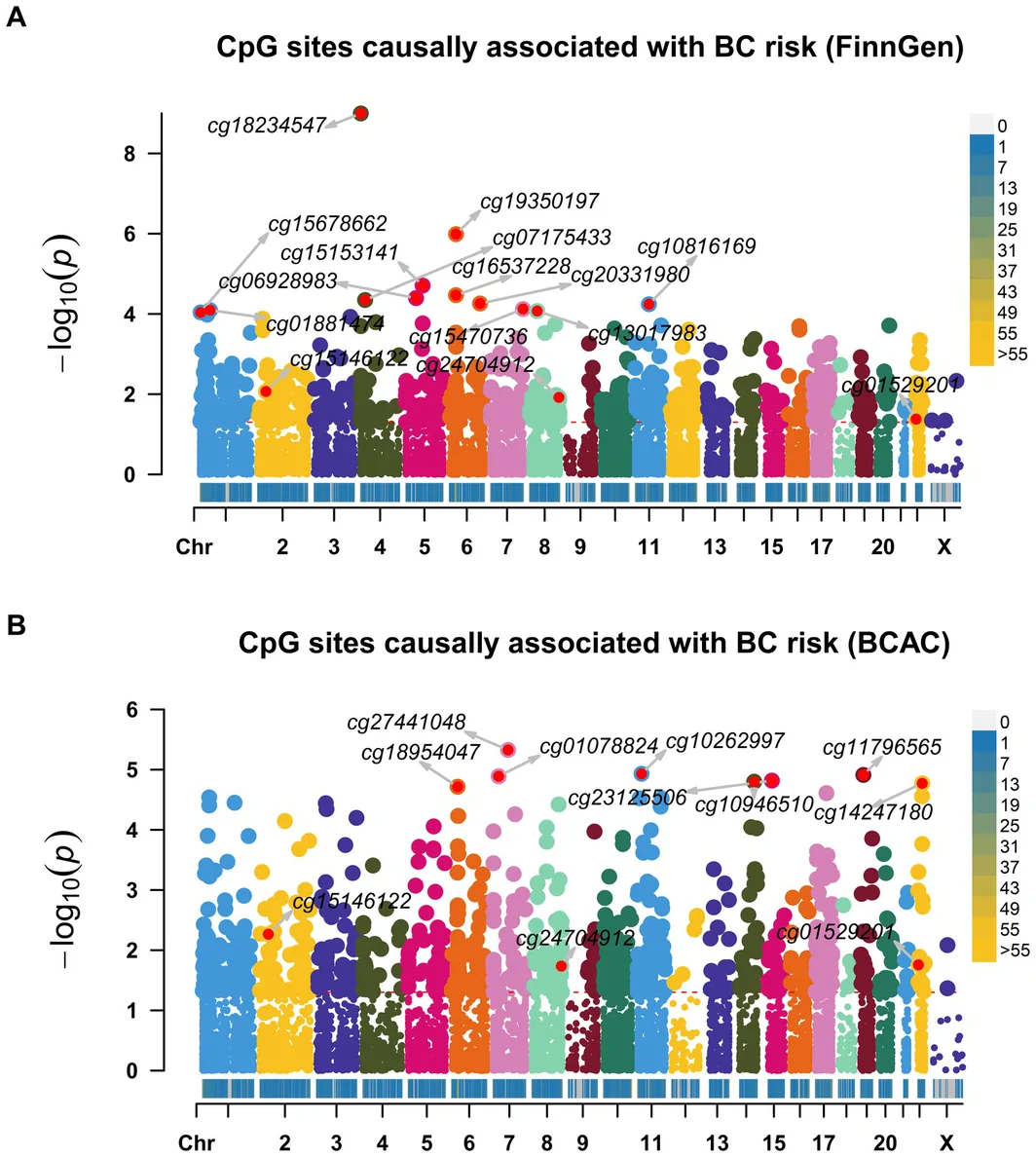
Manhattan plots of MR analysis assessing causal associations between DNA methylation and breast cancer risk. (A) Manhattan plot showing the associations between CpG sites and BC risk in the FinnGen consortium. The *y*‐axis represents the strength of the association as −log_10_(*p*‐value) from the inverse‐variance weighted MR method. The *x*‐axis shows the genomic positions across chromosomes. Lead CpG sites surpassing this threshold are highlighted. (B) Manhattan plot displaying the associations in the BCAC cohort, under the same analytical parameters. The most significant CpGs associated with BC risk in each cohort are labelled.

### 3.3 Validation of Causal CpG‐BC Associations via GSMR Analysis

To further validate the robustness of the 95 cross‐cohort causal CpG sites identified through TSMR, we conducted a GSMR analysis. This method incorporates a heterogeneity‐based model selection procedure (the HEIDI outlier test); as detailed in the Methods, the HEIDI‐outlier filtering did not exclude any instrument at the threshold used here, and GSMR is therefore reported as an independent replication of the TSMR estimates rather than as an additional pleiotropy filter. Genetic instruments for each CpG were rigorously selected based on stringent criteria: SNP significance (*p* < 5 × 10^−8^), LD clumping (*r* ^2^ < 0.05 within a 10,000 kb window using 1000 Genomes European reference data), and a minimum of five independent IVs per CpG to ensure robust instrument strength, with variants having an F‐statistic below 10 being excluded to avoid weak instrument bias. Among the 95 candidate CpGs, 70 were excluded from the GSMR analysis due to an insufficient number of valid IVs after applying these quality controls. Among the remaining 25 CpGs with adequate IVs, GSMR analysis replicated 13 significant associations (FDR < 0.05) for both FinnGen and BCAC outcomes, demonstrating a high consistency with the initial TSMR findings and underscoring the reliability of these specific causal relationships. The effect directions (odds ratios, ORs) for these 13 CpGs were consistent between the GSMR and TSMR analyses. Crucially, eight CpGs demonstrated concordant causal effects across both independent cohorts, highlighting their high confidence as robust risk modifiers. The concordant results from TSMR and GSMR for these eight key CpGs were visualized using a forest plot (Figure 2A), which effectively compares the effect estimates and confidence intervals from both methods, underscoring the reliability of the findings. The complete results, including beta coefficients, standard errors, and *p*‐values for all analysed CpGs, are detailed in Table S4.

**FIGURE 2:**
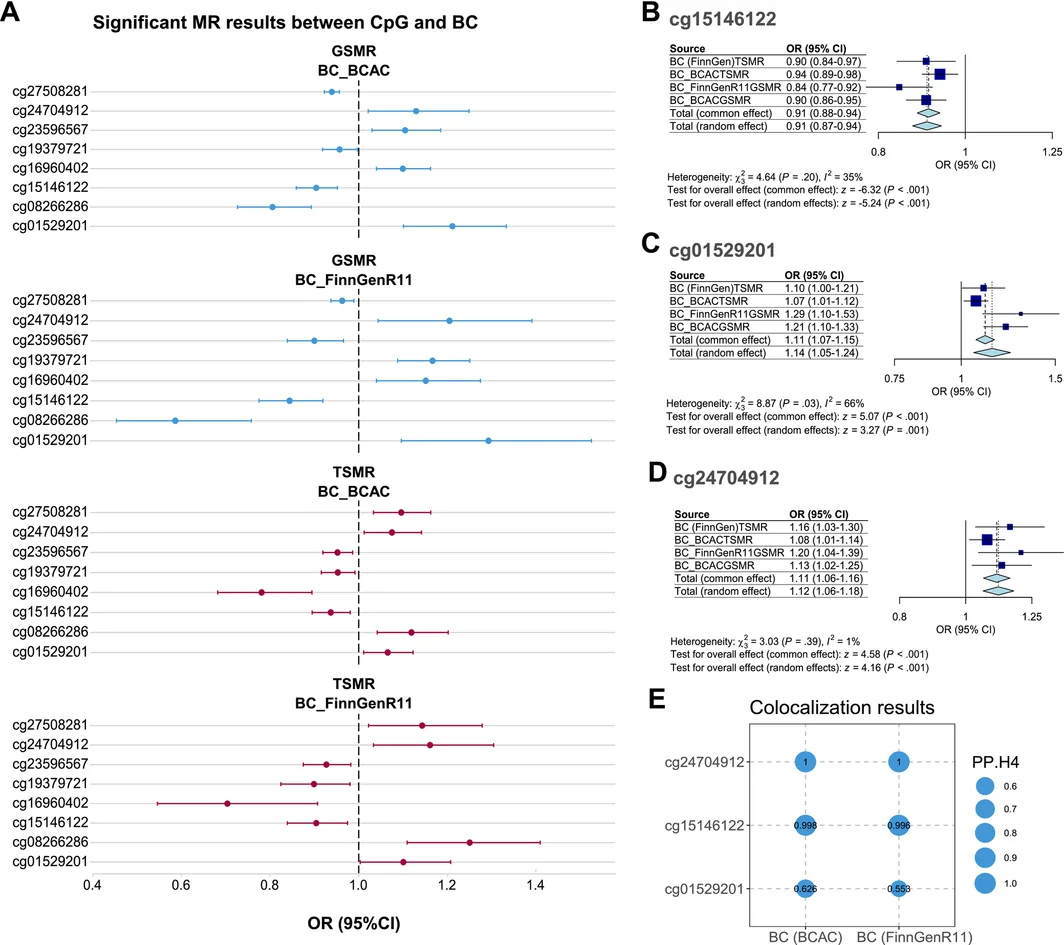
Validation and integration of causal CpG‐breast cancer associations. (A) Forest plot displaying consistent causal effects of 8 CpG sites on BC risk using TSMR and GSMR methods across FinnGen and BCAC cohorts. Odds ratios (ORs) and 95% confidence intervals are shown. (B–D) Meta‐analysis forest plots for three high‐confidence CpGs: cg15146122 (B), cg01529201 (C), and cg24704912 (D), integrating FinnGen and BCAC estimates. (E) Dot plot comparing Bayesian colocalization evidence (PP.H4) for three CpGs across FinnGen and BCAC, with PP.H4 > 0.8 indicating shared causal variants.

### 3.4 Meta‐Analysis Integration of Multi‐Method Causal Evidence

We performed a fixed/random‐effects meta‐analysis to synthesize causal estimates for the eight high‐confidence CpGs identified by both TSMR and GSMR across FinnGen and BCAC cohorts. Three CpGs demonstrated robust, statistically significant associations with BC risk: cg15146122 (OR = 0.91, 95% CI: 0.88–0.94; common‐effect model, *p* = 2.1 × 10^−8^) exhibited a protective effect (Figure 2B). cg01529201 (OR = 1.14, 95% CI: 1.05–1.24; random‐effects model, *I* ^2^ = 43%, *p* = 0.001, Figure 2C), cg24704912 (OR = 1.11, 95% CI: 1.06–1.16; common‐effect model, *p* = 3.8 × 10^−6^, Figure 2D). Notably, all three CpGs showed consistent directionality across TSMR and GSMR methods. Forest plots (Figure 2B–D) visualize the harmonized effect sizes, underscoring their reliability as epigenetic risk modulators. Five CpG sites were not statistically significant (*p* ≥ 0.05): cg08266286 (OR = 0.91, 95% CI: 0.66–1.26; random‐effects model, *I* ^2^ = 95%, *p* < 0.001, Figure S1A), cg16960402 (OR = 0.93, 95% CI: 0.74–1.17; random‐effects model, *I* ^2^ = 91%, *p* = 0.001, Figure S1B), cg19379721 (OR = 0.99, 95% CI: 0.89–1.10; random‐effects model, *I* ^2^ = 90%, *p* < 0.001, Figure S1C), cg23596567 (OR = 0.97, 95% CI: 0.89–1.05; random‐effects model, *I* ^2^ = 85%, *p* < 0.001, Figure S1D) and cg27508281 (OR = 1.02, 95% CI: 0.93–1.12; random‐effects model, *I* ^2^ = 91%, *p* < 0.001, Figure S1E).

### 3.5 Genetic Colocalization Validates Causal Methylation‐BC Links

Colocalization analysis was performed for all three CpGs; strong evidence of a shared causal variant was obtained for two of them. Strong evidence supported shared causal SNPs for cg15146122 (rs440155, PP.H4 = 0.99) and cg24704912 (rs7465455, PP.H4 = 0.99), whereas cg01529201 showed insufficient evidence (PP.H4 < 0.8). Critically, our study provides the first evidence that these SNPs drive BC pathogenesis through potential methylation‐specific mechanisms. These findings redefine known genetic variants as BC‐specific epigenetic drivers, offering new therapeutic targets. Figure 2E visualizes PP.H4 comparisons across CpGs. The complete colocalization results, including shared causal variants for the three CpGs tested, are detailed in Table S5.

### 3.6 PheWAS Reveals Minimal Pleiotropic Effects for Causal CpG Instruments

We performed a PheWAS on the lead SNPs for the three causal CpGs to systematically assess potential horizontal pleiotropy and identify any off‐target effects, which is critical for validating the specificity of the Mendelian randomization findings. The analysis was conducted applying a significance threshold of *p* < 1 × 10^−5^ to account for multiple testing. Reassuringly, the IV for cg15146122 (rs440155) exhibited no significant associations with any of the tested phenotypes, supporting its validity as a specific instrument for BC risk with minimal confounding pleiotropic pathways. In contrast, the IV for cg01529201 (rs595224) showed significant associations with traits including Acute prostatitis and Adverse drug events and drug allergies. Furthermore, the IV for cg24704912 (rs7465455) was significantly associated with Ovarian dysfunction and Neuralgia, neuritis and radiculitis NOS. These pleiotropic associations for rs440155, rs595224 and rs7465455 are illustrated in the Manhattan plots (Figure 3A–C). The presence of these specific off‐target phenotypes suggests that the causal estimates for their respective CpGs may be influenced by distinct biological pathways beyond BC, highlighting the importance of considering such effects in the interpretation of the MR results.

**FIGURE 3:**
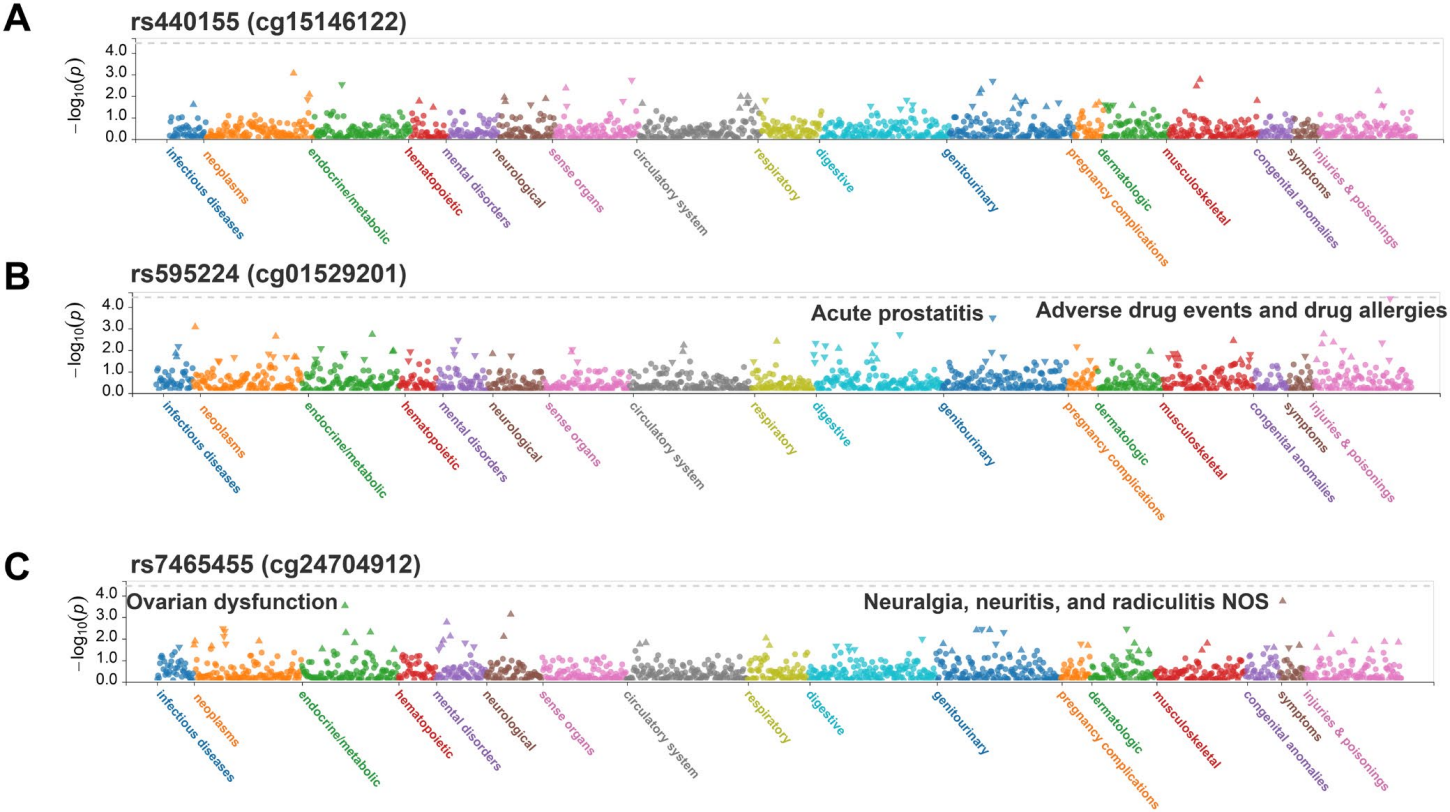
Phenome‐wide association study (PheWAS) of instrumental variables for three causal CpG sites. Manhattan plots display associations between each IV and diverse phenotypes. The *y*‐axis represents the strength of association as −log_10_(*p*‐value); the *x*‐axis shows disease categories. Grey lines indicate the significance threshold (*p* < 1 × 10^−5^). (A) rs440155 (for cg15146122) shows no significant associations, supporting its specificity. (B) rs595224 (cg01529201) associates with acute prostatitis and adverse drug events. (C) rs7465455 (cg24704912) links to ovarian dysfunction and neuralgia.

### 3.7 Protein‐Mediated Pathways Linking CpG Methylation to BC Risk

To investigate the potential mediating role of plasma proteins in the causal pathway between CpG methylation and BC, we conducted a comprehensive TSMR analysis. Initially, we assessed the causal effects of 2940 plasma proteins on BC risk, identifying 203 proteins with significant associations (IVW FDR < 0.05). We then explored the effects of the three key CpG sites on these proteins. The cg01529201 locus was found to influence the expression levels of 41 plasma proteins, with CD207 (*p* = 0.0003) being the most significant hit (Figure 4A). Functional enrichment analysis of these proteins revealed their primary involvement in the activation of immune response (Biological Process), localization to the external side of the plasma membrane (Cellular Component), carbohydrate binding (Molecular Function), and the C‐type lectin receptor signalling pathway (KEGG) (Figure 4B).

**FIGURE 4:**
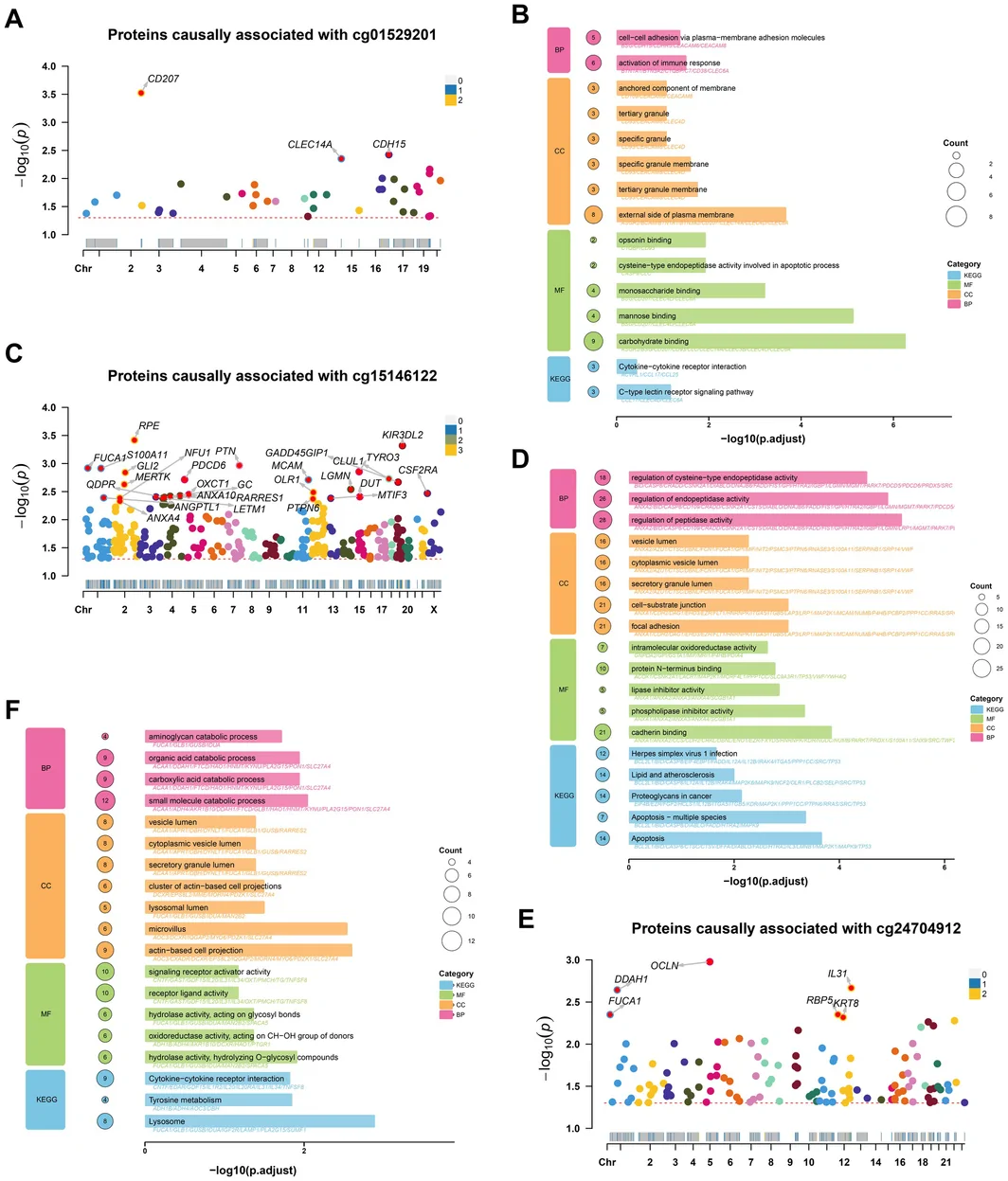
Proteomic mediation and functional enrichment analysis for three causal CpG loci. (A, C, E) Manhattan plots showing plasma proteins causally associated with cg01529201 (A), cg15146122 (C), and cg24704912 (E). Top associations: CD207 (A), RPE (C), OCLN (E). Red line: *p* < 0.05. (B, D, F) Bar plots displaying enriched pathways (top 5 per category; *p*.adjust < 0.05) for proteins linked to each CpG. Colours denote functional categories (BP/CC/MF/KEGG). Dot size indicates gene count.

The cg15146122 locus demonstrated a broader effect, significantly associated with 314 plasma proteins. RPE (*p* = 0.0004) was the most notably influenced protein (Figure 4C). The functional profile of these proteins highlighted enrichment for terms such as the regulation of peptidase activity (BP), focal adhesion (CC), cadherin binding (MF) and the Apoptosis pathway (KEGG) (Figure 4D). For the third locus, cg24704912, analyses indicated a causal effect on 126 proteins, with OCLN (*p* = 0.001) being the top association (Figure 4E). These proteins were predominantly enriched in small molecule catabolic processes (BP), actin‐based cell projections (CC), hydrolase activity acting on O‐glycosyl compounds (MF) and the Lysosome pathway (KEGG) (Figure 4F).

The complete results of the MR analyses between the significant CpG sites and individual plasma proteins are documented in Table S6, while the MR results assessing the causal relationships between all proteins and BC (using BCAC data) are provided in Table S7. This multi‐step analysis delineates specific plasma proteins that potentially mediate the effects of differential methylation at these three CpG loci on BC pathogenesis, uncovering key immune, adhesive and metabolic pathways.

### 3.8 Integrated Alluvial Map Illustrates Mediation Pathways Linking CpG Sites to BC Through Plasma Proteins

Our comprehensive mediation analysis delineated the causal pathways connecting three key CpG sites to BC risk via specific plasma proteins, with the complex network visualized in an alluvial diagram (Figure 5). The analysis established that cg01529201 (IV: rs595224) exerts its effect on BC primarily through the mediator protein ANXA10. In contrast, cg15146122 (IV: rs440155) influenced BC risk via a broader network of 13 mediator proteins, including ALPI, ANXA10, ANXA4, BRK1, FGF2, HTRA2, IL6ST, LGALS1, MECR, OLR1, PLCB2, RARRES1 and TMED8, highlighting a more complex regulatory mechanism. Meanwhile, the effect of cg24704912 (IV: rs7465455) was mediated specifically by the protein ANGPTL2. This integrated mapping provides a holistic view of the epigenetic‐protein‐disease axis, underscoring both shared and distinct mediation pathways through which DNA methylation disparities potentially contribute to BC pathogenesis.

**FIGURE 5:**
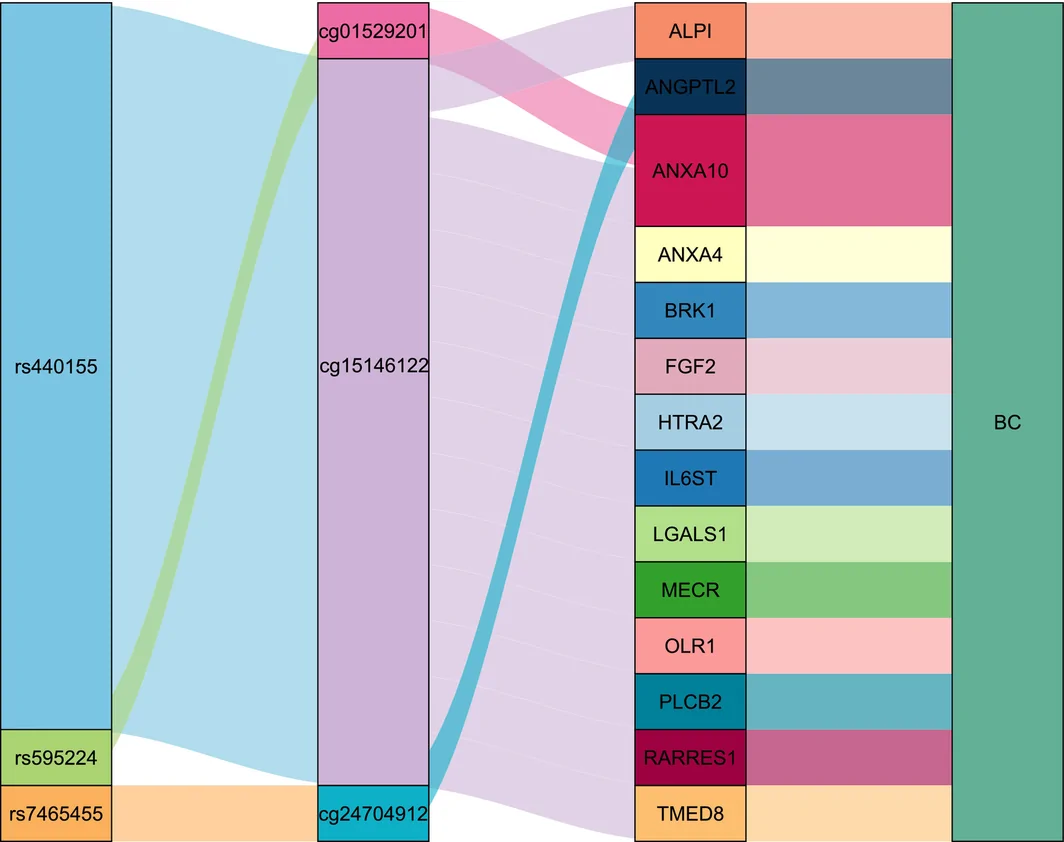
Alluvial diagram illustrating the mediation pathways connecting three key CpG sites to breast cancer risk via plasma proteins. The analysis shows cg01529201 primarily influences BC through ANXA10, cg15146122 via a network of 13 proteins (e.g., FGF2, OLR1) and cg24704912 specifically through ANGPTL2, revealing distinct epigenetic‐protein‐disease axes.

The mediation effect of each CpG site on BC risk was calculated as the product of its effect on the mediator protein (beta1) and the protein's effect on BC (beta2). Confidence intervals (CIs) for these indirect effects were derived using the RMediation package in R. Effects with 95% CIs excluding zero were deemed statistically significant. The complete outcomes, including effect sizes and CI ranges, are summarized in Table 1.

**TABLE 1:** Two‐step mediation MR analysis linking the three causal CpG sites to breast cancer risk through plasma proteins. Twenty‐five CpG‐protein pairs were evaluated; in 15 of them the 95% confidence interval of the indirect effect excluded zero (bold mediator names; 14 distinct proteins).

IV	Exposure	Mediator	Outcome	Beta1	Beta2	Mbeta	Lo_CI	Up_CI
rs440155	cg15146122	**ALPI**	BC	0.06	−0.05	−0.003	−0.01	−1.50E‐04
rs440155	cg15146122	**ANXA10**	BC	0.09	0.39	0.04	0.01	0.07
rs440155	cg15146122	**ANXA4**	BC	0.09	−0.57	−0.05	−0.10	−0.01
rs440155	cg15146122	BRAP	BC	0.07	0.31	0.02	−1.36E‐03	0.05
rs440155	cg15146122	**BRK1**	BC	0.07	−0.09	−0.01	−0.01	−1.30E‐04
rs440155	cg15146122	DTD1	BC	0.07	−0.07	−0.005	−0.01	3.99E‐04
rs440155	cg15146122	**FGF2**	BC	0.07	−0.05	−0.003	−0.01	−4.07E‐04
rs440155	cg15146122	**HTRA2**	BC	0.07	−0.30	−0.02	−0.05	−6.86E‐05
rs440155	cg15146122	**IL6ST**	BC	0.08	−0.05	−0.004	−0.01	−1.35E‐06
rs440155	cg15146122	**LGALS1**	BC	0.06	0.07	0.005	2.88E‐04	0.01
rs440155	cg15146122	**MECR**	BC	0.08	−0.14	−0.01	−0.03	−6.87E‐04
rs440155	cg15146122	MIF	BC	0.07	−0.15	−0.01	−0.03	2.88E‐04
rs440155	cg15146122	OGA	BC	0.06	0.13	0.01	−1.31E‐04	0.02
rs440155	cg15146122	**OLR1**	BC	0.09	−0.12	−0.01	−0.02	−2.10E‐03
rs440155	cg15146122	PDIA4	BC	0.06	−0.14	−0.01	−0.03	8.59E‐04
rs440155	cg15146122	**PLCB2**	BC	0.07	0.33	0.02	5.61E‐05	0.05
rs440155	cg15146122	**RARRES1**	BC	−0.09	−0.05	0.004	6.67E‐04	0.01
rs440155	cg15146122	SRP14	BC	0.07	0.26	0.02	−1.77E‐04	0.05
rs440155	cg15146122	**TMED8**	BC	0.06	−0.27	−0.02	−0.04	−2.61E‐04
rs7465455	cg24704912	ACAA1	BC	−0.08	−0.10	0.01	−3.19E‐04	0.02
rs7465455	cg24704912	**ANGPTL2**	BC	−0.08	0.06	−0.01	−0.01	−2.68E‐04
rs7465455	cg24704912	FUOM	BC	−0.08	−0.07	0.01	−4.04E‐04	0.01
rs7465455	cg24704912	RARRES2	BC	−0.08	0.06	−0.005	−0.01	4.45E‐04
rs595224	cg01529201	**ANXA10**	BC	0.08	0.39	0.03	3.22E‐03	0.07
rs595224	cg01529201	CLEC3B	BC	0.07	−0.07	−0.005	−0.01	4.61E‐04

Abbreviations: BC, breast cancer; Beta1, effect size of CpG on protein; Beta2, effect size of protein on BC; IV, instrumental variable; Lo_CI, lower 95% confidence interval; Mbeta, the mediating effect value of CpG on BC (beta1*beta2); Up_CI, upper 95% confidence interval. Significance is indicated solely by the bold mediator names: the 15 CpG‐protein pairs whose 95% confidence interval of the indirect effect excluded zero (14 distinct proteins).

### 3.9 Chromosomal Mapping of CpG Sites and Their Mediator Proteins Using PhenoGram

We precisely mapped the genomic locations of the three causal CpG sites and their downstream mediator proteins using PhenoGram to visualize their spatial relationships. Specifically, cg01529201 on chromosome 22 (22q11.21, chr22:20140202–20140202, the intron region of ENSG00000299199) was linked to its mediator ANXA10 on chromosome 4 (4q32.3, chr4:169013688–169108887) (Figure 6A), while cg24704912 on chromosome 8 (8q24.3, chr8:142427421–142427421, the exon region of PTP4A3) was linked to its mediator ANGPTL2 on chromosome 9 (9q33.3, chr9:129849627–129884914) (Figure 6C), indicating potential distal trans‐regulatory mechanisms. In contrast, cg15146122 on chromosome 2 (2p22.1, chr2:40472773–40472773, the intron region of SLC8A1) demonstrated a complex trans‐regulatory network influencing 13 mediator proteins distributed across multiple chromosomes, including ALPI on chromosome 2 (2q37.1, chr2:233320835–233325463) and FGF2 on chromosome 4 (4q27‐q28.1, chr4:123747837–123819391) (Figure 6B). This chromosomal cartography provides crucial spatial context for the epigenetic‐proteomic causal pathways, highlighting both cis‐acting and trans‐acting regulatory architectures underlying BC susceptibility.

**FIGURE 6:**
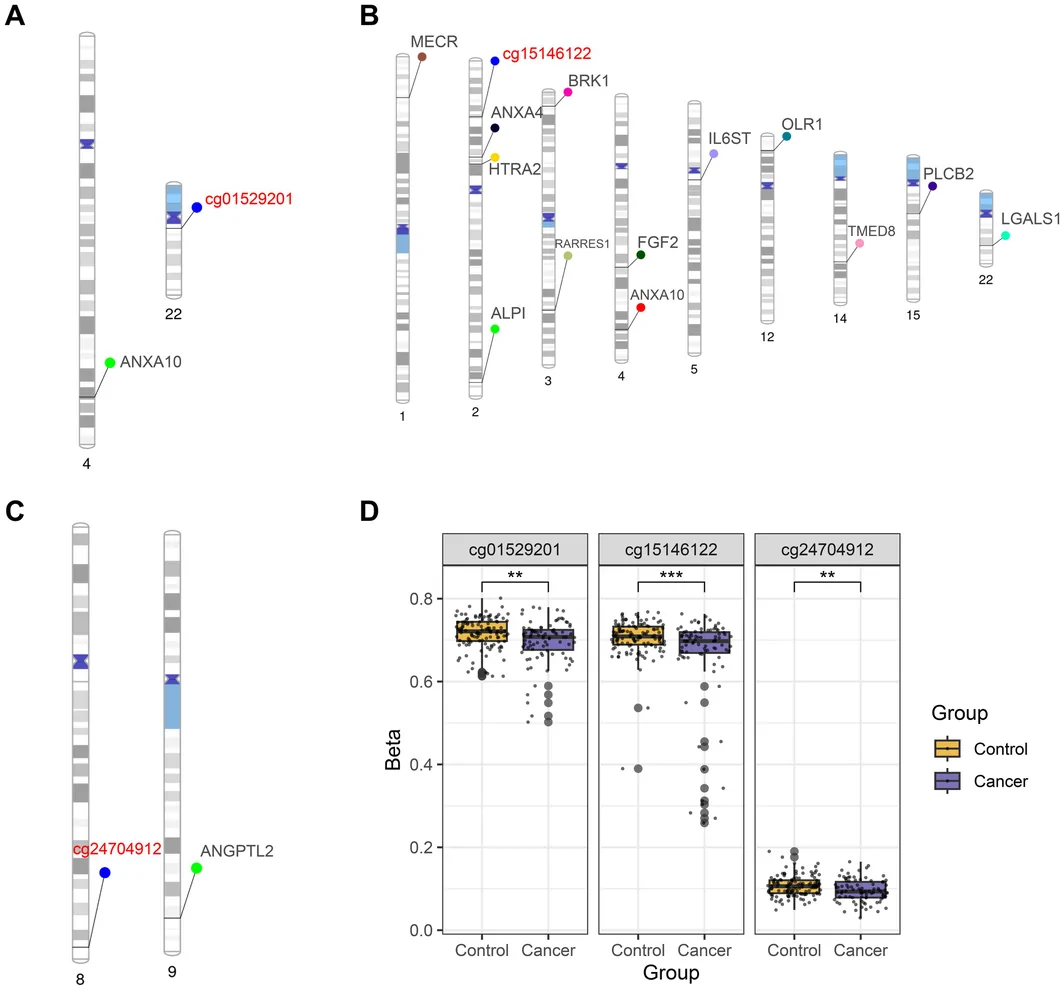
Genomic mapping of causal CpG sites and validation of differential methylation. (A–C) Chromosomal locations of three CpG sites (cg01529201, cg15146122 and cg24704912) and their mediator proteins (e.g., ANXA10, ANGPTL2, FGF2) mapped using PhenoGram, revealing trans/cis regulatory architectures. (D) Box plots confirm significant hypomethylation at all three CpGs in BC patients versus controls, supporting their biomarker potential for early detection. **p < 0.01, ***p < 0.001.

### 3.10 Differential Methylation of Three CpG Sites in BC Patients vs. Healthy Controls

Box plot analysis (Figure 6D) visually compared the methylation levels (beta values) of three CpG sites between BC patients and healthy controls, demonstrating distinct methylation patterns. All three CpG sites exhibited significantly reduced methylation levels in BC patients compared to controls. This consistent hypomethylation across all loci suggests a potential role for epigenetic dysregulation in BC pathogenesis. The observed differential methylation underscores the value of these CpG sites as candidate biomarkers for further validation in early detection or monitoring frameworks.

### 3.11 Single‐Cell Transcriptomic Profiling Reveals Cell‐Type‐Specific Expression of Mediator Proteins in BC

We characterized the cellular expression patterns of 14 mediator proteins implicated in BC pathogenesis using single‐cell RNA sequencing data processed via the Seurat pipeline. The analysis incorporated rigorous quality control, including doublet removal with DoubletFinder and batch‐effect correction using Harmony, in which the five receptor‐status groups (normal, ER+, HER2+, PR+ and TNBC) were treated as the integration batches rather than individual donors. UMAP visualization identified seven distinct cell types within the tumour microenvironment: epithelial cells, endothelial cells, fibroblasts, B cells, T cells, macrophages and dendritic cells (Figure 7A). Dot plot analysis demonstrated that 13 mediator‐encoding genes (excluding ALPI, which was not detected in the dataset) exhibited cell‐type‐specific expression, with pronounced differences between normal and diseased states (Figure 7C). Notably, FGF2 and OLR1 showed significant differential expression across multiple cell populations, highlighting their potential roles in BC progression. UMAP visualizations confirmed the distinct distribution of FGF2 and OLR1 expression within BC subtypes (Figure 7B), underscoring their relevance to tumour heterogeneity. These findings provide a high‐resolution map of mediator protein dynamics, offering insights into their cell‐type‐specific functions in BC pathogenesis.

**FIGURE 7:**
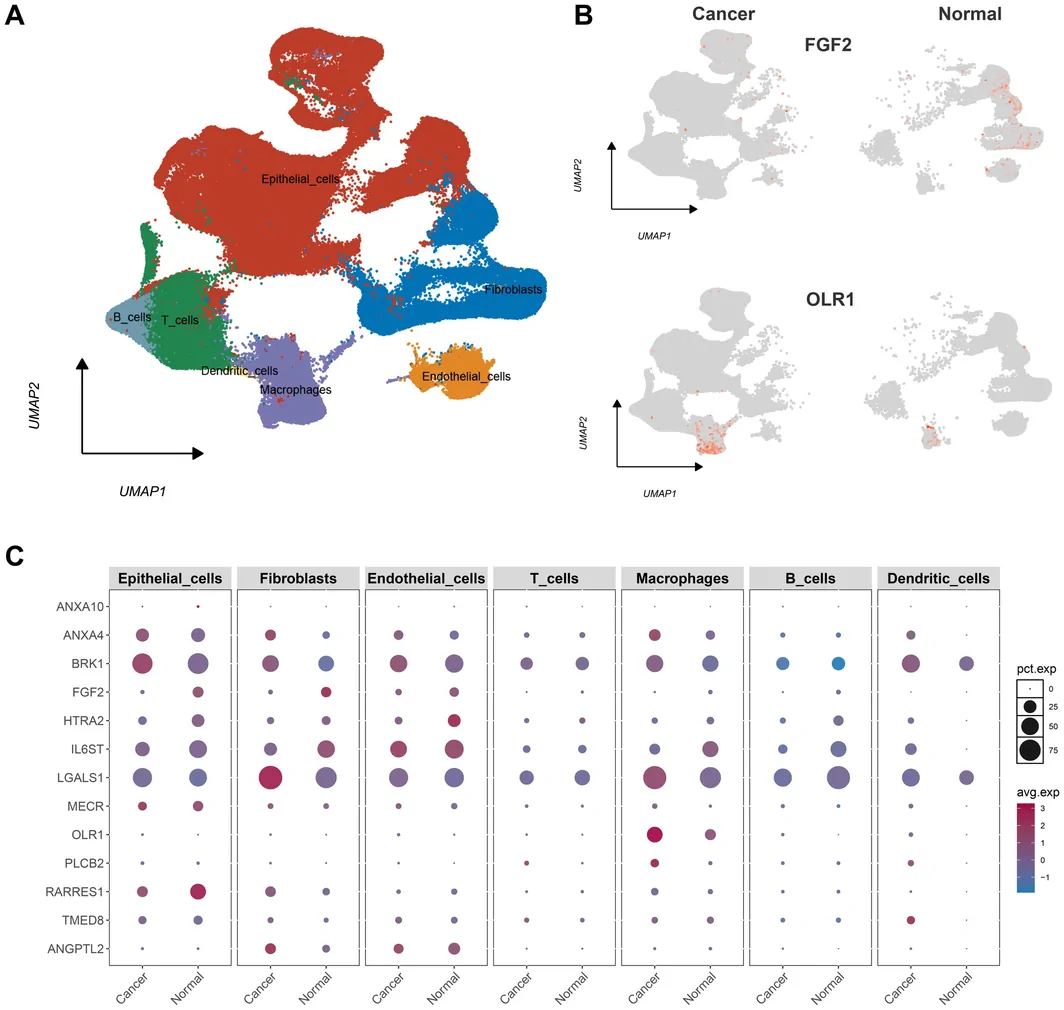
Single‐cell transcriptomic analysis of mediator protein expression in the breast tumour microenvironment. (A) Single‐cell RNA sequencing data processed through the Seurat workflow with doublet removal (DoubletFinder) and batch correction (Harmony) revealed seven distinct cell types via UMAP visualization. (B) UMAP plots show distinct expression distributions of key mediators FGF2 and OLR1 across BC subtypes. (C) Dot plot analysis illustrates cell‐type‐specific expression of 13 mediator‐encoding genes (excluding ALPI) in normal versus diseased states, highlighting FGF2 and OLR1 as multi‐population regulators of BC progression.

## 4 Discussion

This multi‐omics Mendelian randomization study provides robust, MR‐supported evidence for a potential causal role of blood‐based DNA methylation in BC pathogenesis. Through a comprehensive analytical framework integrating methylation quantitative trait loci from the GoDMC consortium and large‐scale GWAS data from both BCAC and FinnGen, we identified 95 CpG sites with MR‐supported associations with BC risk, of which 8 were concordant between TSMR and GSMR. Meta‐analysis confirmed three high‐confidence loci, including cg15146122, which demonstrated a protective effect, and two risk‐enhancing CpGs: cg01529201 and cg24704912. Bayesian colocalization provided strong evidence for shared causal variants for two of these CpG sites, while proteome‐wide MR analysis revealed extensive mediation through plasma proteins, with the three key CpGs influencing 41‐314 proteins.

Prior evidence on the causal epigenetics of BC remains limited. To our knowledge, only a limited number of DNA methylation sites have been examined for causal associations with BC risk, predominantly exposure‐related loci such as smoking‐associated (e.g., AHRR) and alcohol‐associated methylation marks investigated by MR [10, 11], together with heritable methylation marks linked to BC susceptibility [5]. No previous study has reported cg15146122, cg01529201 or cg24704912 as causally associated with BC—our three high‐confidence loci are therefore novel. For circulating proteins, several proteome‐wide MR studies have implicated a limited set of plasma proteins in BC risk, but none have positioned them as mediators downstream of specific methylation loci within an integrated causal framework. Our study extends the field by systematically linking blood methylation, plasma proteomics and BC risk.

Our findings align with and extend the growing evidence that blood‐based DNA methylation changes may serve as early indicators of cancer development [6]. The identification of 95 CpG sites with MR‐supported associations with BC risk (TSMR results) reinforces the potential of DNA methylation biomarkers for cancer risk prediction, consistent with prospective studies showing methylation alterations preceding clinical diagnosis [12]. The protective association of cg15146122 hypermethylation, despite its location on chromosome 2 rather than near established tumour suppressor genes, suggests complex trans‐acting mechanisms in epigenetic regulation. This finding parallels emerging research showing that methylation sites can influence gene expression through long‐range chromosomal interactions and three‐dimensional genome architecture [37]. Similarly, the risk‐enhancing effects of cg01529201 and cg24704912 hypermethylation support the classical model of epigenetic silencing of growth regulators, while their specific genomic locations indicate potentially novel regulatory mechanisms in BC pathogenesis.

The mediator proteins identified through our proteome‐wide analysis play particularly compelling roles in cancer biology. For instance, FGF2 (fibroblast growth factor 2), which showed strong association with cg15146122, promotes angiogenesis and tumour progression through activation of MAPK and PI3K‐AKT pathways [38]. Beyond these well‐established roles, recent evidence suggests FGF2 may also contribute to immunotherapy resistance by promoting immunosuppressive microenvironments in breast tumours [39]. Similarly, OLR1 (oxidized low‐density lipoprotein receptor 1), associated with cg24704912, facilitates oxidized LDL uptake in tumour cells, promoting metastasis and chemoresistance in BC models [40]. The involvement of ANXA10, linked to cg01529201, is particularly noteworthy as it regulates apoptosis and cellular differentiation [40] in hormone‐responsive tissues, suggesting potential mechanisms for its role in BC development. The functional enrichment patterns observed among these mediator proteins provide additional mechanistic insights into how DNA methylation differences influence BC risk through immune response regulation, apoptosis and metabolic pathways.

The major strengths of our study include the use of the largest available meQTL summary statistics from the GoDMC consortium, the replication of findings across two independent BC GWAS datasets, and the integration of single‐cell transcriptomic data to characterize cellular expression patterns of mediator proteins within the tumour microenvironment. The application of multiple sensitivity analyses, including MR‐Egger regression, Cochran's *Q* test and MR‐PRESSO [29], strengthens the validity of our causal inferences. However, several limitations should be acknowledged. The primary focus on European‐ancestry populations limits the generalizability of our findings to other ethnic groups. While MR design reduces concerns about reverse causation, residual confounding due to unbalanced pleiotropy cannot be completely excluded. The mediation analysis assumes linear relationships in the CpG‐protein‐disease pathway, though biological systems often exhibit more complex, non‐linear dynamics. Finally, while we identified several promising candidate proteins, functional validation through experimental models is needed to establish definitive mechanistic causality.

The clinical implications of our work are substantial, particularly in the context of BC prevention and early detection. The identified CpG sites and their associated plasma protein mediators represent promising candidates for biomarker development, potentially enabling improved risk stratification beyond traditional factors such as family history and genetic predisposition markers. The cell‐type‐specific expression patterns of mediator proteins revealed by single‐cell RNA sequencing provide insights into which cellular compartments may be most relevant for targeted interventions. Future research should prioritize validation of these findings in diverse populations, functional characterization of the identified CpG sites and mediator proteins using experimental models, and assessment of their utility in clinical settings. Longitudinal studies examining the dynamics of methylation changes in relation to cancer development will be particularly valuable. By bridging population genetics with molecular mechanisms, our study provides a foundation for developing more effective prevention strategies and targeted therapies for BC.

## 5 Conclusion

In conclusion, this comprehensive multi‐omics investigation provides robust and novel evidence supporting the causal role of blood‐based DNA methylation in BC pathogenesis. The application of a two‐sample Mendelian randomization framework, validated across two independent large‐scale cohorts (BCAC and FinnGen), coupled with extensive sensitivity analyses and Bayesian colocalization, ensures the reliability of our causal inferences against confounding and reverse causation. Our study newly identifies three high‐confidence CpG sites—cg15146122 (protective), cg01529201 (risk‐enhancing) and cg24704912 (risk‐enhancing)—as key epigenetic determinants of BC risk, and further reveals that their effects are mediated through 15 CpG‐protein pairs involving 14 distinct plasma proteins implicated in immune regulation, apoptosis and metabolic pathways. The integration of single‐cell transcriptomics precisely localized these mediator proteins within specific cellular compartments of the tumour microenvironment, providing unprecedented resolution into the cell‐type‐specific mechanisms underlying epigenetic regulation of BC development.

Despite these strengths, several limitations should be acknowledged. Our findings are primarily based on European‐ancestry populations, which may limit the generalizability to other ethnic groups with different genetic architectures and environmental exposures. While MR reduces reverse causation concerns, residual confounding from unmeasured environmental factors cannot be entirely excluded. The mediation analysis assumes linear relationships in the CpG‐protein‐disease pathway, though biological systems may exhibit more complex, non‐linear dynamics. Additionally, although we identified promising candidate biomarkers, functional validation through experimental models is needed to establish definitive mechanistic causality.

Future research should prioritize validation of these epigenetic markers in diverse populations, functional characterization using CRISPR‐based approaches to manipulate specific CpG sites in model systems, and longitudinal studies to track methylation changes during cancer progression. The integration of multi‐omics data with clinical outcomes could enable personalized risk prediction models, while organoid systems and animal models could decipher the direct mechanisms of action. By bridging genetic epidemiology with functional insights, our work lays the foundation for developing epigenetic‐based strategies for BC prevention, early detection, and targeted therapy, ultimately contributing to precision oncology approaches.

## Author Contributions

**Wei Wang:** software, formal analysis, visualization. **Ziye Cheng:** validation, writing – original draft. **Ruidong Liu:** data curation. **Xufeng Cheng:** conceptualization, funding acquisition. **Hanghang Chen:** validation, methodology, writing – review and editing, funding acquisition.

## Funding

This work was supported by the National Project for Leading Traditional Chinese Medicine Specialties (grant no. CZ0338‐03); the Henan Province Traditional Chinese Medicine Key Discipline Construction Project (grant no. CZ0366‐07); and the Doctoral Research Foundation of the First Affiliated Hospital of Henan University of Chinese Medicine (grant no. 2024BSJJ044).

## Disclosure

Code availability: All analysis code (R scripts) used in this study is openly available at https://github.com/hanghang20220428/DMP_MR_BC. The repository contains the complete analysis pipeline in reproducible order, together with the single‐cell quality‐control thresholds, software versions, and random seeds described above.

## Ethics Statement

All studies were approved by their corresponding ethics review boards, and all subjects provided informed consent. This study used publicly available GWAS summary statistics data without individual information, and thus no ethical approval was required.

## Consent

This study has been approved by all authors for publication.

## Conflicts of Interest

The authors declare no conflicts of interest.

## Supporting information


**Figure S1:** Forest plots of meta‐analysis for five hypomethylated CpG sites. Panels (A–E) display the results of inverse‐variance weighted meta‐analyses for CpG sites cg08266286, cg16960402, cg19379721, cg23596567 and cg27508281, respectively. The results for these five CpG sites were not statistically significant (*p* ≥ 0.05). Each plot shows the effect size for individual studies, along with the overall pooled effect estimate and its 95% confidence interval.

**Table S1:** Data sources.
**Table S2:** Differential DNA methylation sites in blood samples between control and breast cancer.
**Table S3:** TSMR results between CpG sites and breast cancer.
**Table S4:** GSMR results between significant CpG sites (TSMR) and breast cancer.
**Table S5:** Colocalization results.
**Table S6:** MR results between significant CpG sites and each plasma protein.
**Table S7:** MR results between each plasma protein and BC risk (BCAC).

## Data Availability

The data that support the findings of this study are openly available from the sources listed below; no new primary data were generated. All data sources, original publications and accession numbers used in this study are detailed in Table S1. The breast cancer and methylation quantitative trait locus summary statistics are publicly available from the Breast Cancer Association Consortium, FinnGen (release 11), the Genetics of DNA Methylation Consortium and the UK Biobank Pharma Proteomics Project under their respective data access policies. The methylation array data (GSE104942) and the single‐cell RNA sequencing data (GSE161529) analysed during this study are available from the Gene Expression Omnibus. All data generated during this study are included in this published article and its supplementary information files (Tables S2–S7). The R analysis code is openly available at https://github.com/hanghang20220428/DMP_MR_BC.
