# Supplementary material for: Integrated Multi‐Omics Analyses Reveal Causal DNA Methylation‐Breast Cancer Associations Mediated by Plasma Proteins
Source: J Cell Mol Med. 2026 Nov 8;30(21):e71424. doi: 10.1111/jcmm.71424 (PMC13746094; doi:10.1111/jcmm.71424)
Supplement: Supplementary file 1 [file JCMM-30-e71424-s002.pdf]

Figure S1

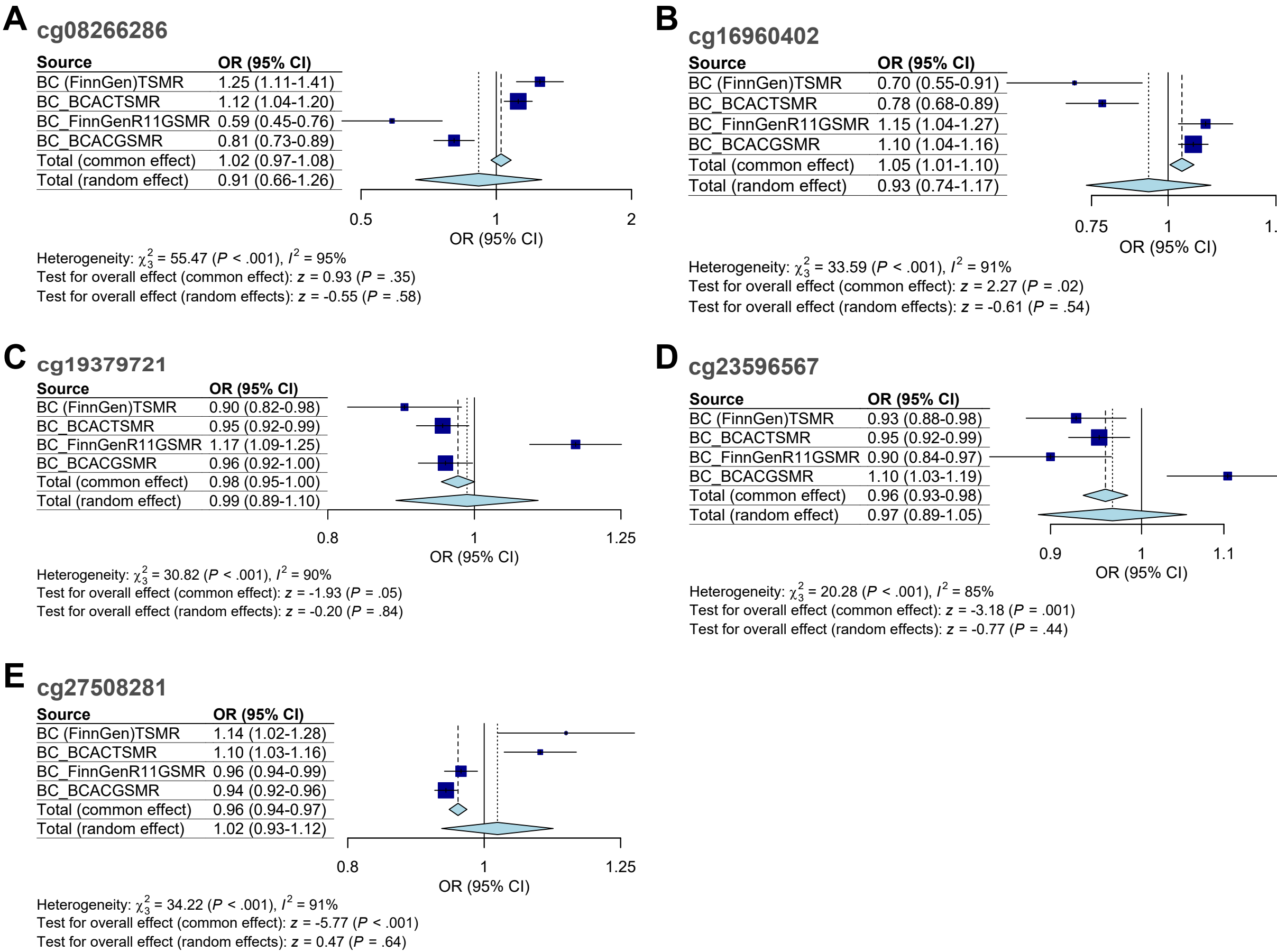

Figure S1 Forest plots of meta-analysis for five hypomethylated CpG sites. Panels (A-E) display the results of inverse-variance weighted meta-analyses for CpG sites cg08266286, cg16960402, cg19379721, cg23596567, and cg27508281, respectively. The results for these five CpG sites were not statistically significant (P ≥ 0.05). Each plot shows the effect size for individual studies, along with the overall pooled effect estimate and its 95% confidence interval
